# CT‐Optimal Stimulation Modulates Somatosensory Processing

**DOI:** 10.1111/psyp.70146

**Published:** 2025-09-08

**Authors:** A. Ribeiro‐Carreira, Márcia da‐Silva, Ana Rita Pereira, Maria Teresa Carrillo‐de‐la‐Peña, Joana Coutinho, Adriana Sampaio, Alberto J. González‐Villar

**Affiliations:** ^1^ Psychological Neuroscience Laboratory (PNL), Research Center in Psychology (CIPsi), School of Psychology Universidade do Minho Braga Portugal; ^2^ Brain and Pain (BaP) Lab, Departamento de Psicoloxía Clínica y Psicobioloxía, Facultade de Psicoloxía Universidade de Santiago de Compostela Santiago de Compostela Spain; ^3^ Instituto de Psicoloxía (IPsiUS), Universidade de Santiago de Compostela Santiago de Compostela Spain

**Keywords:** affective touch, C‐tactile fibers, somatosensation, somatosensory evoked potentials

## Abstract

Touch has an affective dimension, conveyed through low‐threshold mechanoreceptors known as C‐tactile (CT) afferents, which are activated by gentle, caress‐like contact. While there is evidence that these fibers modulate nociceptive input, their influence on the processing of other somatosensory afferent activity remains largely unknown. In this study, we explored how slow brushing (CT‐optimal stimulation) modulates somatosensory evoked potentials (SEPs) elicited by electrical stimulation of the median nerve (occurring at 0.7 to 3.7 s after stimulus onset), compared to vibration (at 200 Hz) and no touch, in 30 healthy participants. CT‐targeted stimulation was delivered using a robotic arm developed in‐house equipped with a cosmetic brush, which applied slow brushing movements at CT‐optimal speeds (~3 cm/s) over the dorsal forearm. Vibrotactile stimulation, targeting A‐beta fibers, was delivered using vibration motors adjacent to the brushed area, with intensity calibrated to match the perceived strength of brushing. SEPs were recorded under these three conditions. Our results showed no differences between slow brushing, vibration, and no touch conditions in the amplitude of early SEPs recorded over the somatosensory cortex (N20, P25, N30, and P45), which may indicate that CT stimulation does not affect early cortical processing of somatosensory information. However, a reduced frontocentral P150 SEP component was observed during slow brushing compared to the other conditions. This finding suggests that CT stimulation may reduce somatosensory input transmitted through the lemniscal system, possibly engaging brain areas involved in attentional and cognitive processing.

## IMPACT STATEMENT

We show that the P150 potential evoked by somatosensory electrical stimulation is reduced when these stimuli are applied during slow brushing. These results extend our understanding of C‐tactile touch beyond its established role in reducing nociceptive input, revealing its influence also on somatosensory input—conveyed by the lemniscal system—and suggesting effects on attentional and cognitive processes.

## 1 Introduction

Touch is a crucial kind of interaction throughout life. Strong affective bonds thrive on close physical touch, making it decisive for our development as social individuals (Cascio et al. 2019; Miguel et al. 2020; Croy et al. 2022; Schirmer et al. 2023). Over the past few decades, a somatosensory system associated with affective touch has been described, involving a subclass of low‐threshold mechanoreceptors C‐fibers (C‐LTMRs), known as C‐tactile or CT fibers (CT) which are essentially found in hairy skin. Microneurography techniques have shown that these unmyelinated mechanosensory afferents encode gentle motion and pressure on the skin, responding optimally to slow‐moving stimuli and human skin temperatures (Olausson et al. 2010; Vallbo et al. 1999; Wessberg et al. 2003). CT fibers terminate in the dorsal horn of the spinal cord, where second‐order neurons project to the thalamus and synapse with third‐order neurons that project to cortical brain regions (Marshall et al. 2019). Specifically, CT‐mediated affective touch has been found to activate cortical brain areas involved in social and emotional processing, such as the posterior insula, the posterior superior temporal sulcus, and the anterior cingulate cortex (Gordon et al. 2013; Morrison 2016a).

CT‐optimal touch has been reported to induce rewarding and pleasurable effects, showing a positive correlation with perceived pleasantness (Ackerley et al. 2014). However, the role of CT fibers in processing other somatosensory signals, including nociceptive stimuli, is less understood. Research has shown that slow brushing can reduce pain, suggesting a modulatory role of CT fibers in nociceptive input (Liljencrantz et al. 2017). This effect has been observed in naturalistic scenarios (Reddan et al. 2020) from early life (Gursul et al. 2018) through adulthood (Meijer et al. 2023; Von Mohr, Crowley, et al. 2018), underscoring its value in comforting individuals in pain or suffering. Yet, the neural mechanisms behind this analgesic effect remain unclear. While the classical gate control theory proposed that activating A‐beta mechanoreceptive fibers near an injury inhibits nociceptive signals from C fibers at the spinal cord level (Melzack and Wall 1965), recent evidence suggests that pain mechanisms are more complex than this simple balance between A‐beta and C fibers (Woolf 2022). Dorsal horn interneurons are hypothesized to integrate slower C‐fiber input with faster A‐beta input. This interaction may enhance the affective aspects of tactile experiences, suggesting that CT input modulates A‐beta signaling to shape the perception of touch beyond its physical properties (Schirmer et al. 2022).

The extent to which CT touch can modulate somatosensory input beyond nociception remains unexplored. In this study, we used somatosensory‐evoked potentials (SEPs)—time‐locked electrophysiological signals following electrical stimulation of the median or tibial nerve (Macerollo et al. 2018). This technique allows us to explore central and peripheral nervous structures, particularly the dorsal somatosensory system, which conveys vibration, discriminative touch, and proprioception (MacDonald et al. 2019), a type of neural information that ascends through the dorsal column and is transmitted via the lemniscal pathway to thalamic structures (Cruccu et al. 2008; MacDonald et al. 2019).

This experiment investigates whether somatosensory input, measured by SEPs and subjective sensation intensity, is modulated by the activation of CT receptors (via a robotic arm with a brush) compared to vibration or no concomitant stimulation. We analyzed both early cortical components—such as N20, P25, N30, and P45—and later components—such as N60 and P150. We hypothesize that the intensity of sensation, ranging from discernable touch to pain, will be lower, and SEP amplitudes will be reduced when electrical stimulation is paired with CT‐targeted stimulation compared to vibration or no stimulation.

## 2 Materials and Methods

### 2.1 Participants

The sample consisted of 30 healthy individuals aged between 18 and 36 (*M* = 20.71; SD = 2.64). All participants were students at the University of Minho and were recruited via the university's credit platform, with participation rewarded through academic credits. Following the guidelines outlined by Lakens (2022) the sample size is justified by taking into account factors such as available resources and the sample sizes reported in previous related studies using EEG and either pain or CT‐touch (within a range between 25 and 32 participants; e.g., Perri et al. 2019; Von Mohr, Crowley, et al. 2018; Von Mohr, Krahé, et al. 2018).

Exclusion criteria included a history of chronic pain and cardiac, psychiatric, or neurological conditions, injured skin on the forearms or hands, pain on the day of the experiment, and substance abuse. Participants were informed about the experimental procedure and provided written informed consent before the experiment began. This study was conducted in accordance with the Declaration of Helsinki and was approved by the Ethics Committee for Social and Human Sciences of the University of Minho (ref. CEICSH 030/2022).

### 2.2 Self‐Report Measures

Before the EEG recording, participants completed several self‐report measures, including a socio‐demographic questionnaire, the Edinburgh Handedness Inventory, and the Patient Health Questionnaire (PHQ‐9) to assess depression levels (Lamela et al. 2020). Additionally, participants completed the Portuguese version of the Touch Experiences and Attitudes Questionnaire (TEAQ) to assess attitudes and experiences related to positive touch (Pereira et al. 2023; Trotter et al. 2018). The TEAQ comprises six subscales that concern Friends and family touch, Current intimate touch, Childhood touch, Attitude to self‐care, Attitude to intimate touch, and Attitude to unfamiliar touch. Participants also completed the NEO Five‐Factor Inventory (NEO‐FFI) to assess personality traits (Costa and McCrae 2012; Magalhães et al. 2014).

### 2.3 Experimental Procedure

Electrical stimuli to elicit somatosensory evoked potentials (SEPs) were delivered over the median nerve of the left wrist under three distinct experimental conditions: SEPs with concomitant brushing (CT‐targeted stimulation), SEPs with concomitant vibration (discriminative touch), and SEPs alone (see Figure S1 for a photograph of the experimental setup).

CT‐targeted Stimulation was administered to the participants' left dorsal forearm using a robotic arm developed in‐house equipped with a cosmetic brush. The brushing movement covered a 14 cm strip of the forearm, applied at CT‐optimal speeds (~3.2 cm/s), and returned to the starting point with each brushing stroke lasting 4.4 s. The robotic arm had 2 degrees of freedom: *X*‐axis and roll. The *X*‐axis was driven by a Nema17 stepper motor (Eleksmaker) controlled by a TMC2209 IC (Bigtreetech), while the roll movement was driven by a hs‐485hb servomotor (Hitec). All the motors were ultimately controlled by an Arduino Nano board. To prevent visual feedback, participants could not see the movement of the robotic arm, as the brush was inside a box.

Discriminative Touch (Vibrotactile) Stimulation targeted A‐beta fibers primarily conveyed through the dorsal column‐medial lemniscus pathway (Caylor et al. 2019). Vibrotactile stimulation was delivered using two linear resonant actuator vibration motors controlled by the DVR2605L haptic module (TexasInstruments) at approximately 200 Hz. The vibration motors used were “Vybronics VG0832029D”. The noise generated by these small motors is relatively low (50 dB Max.) at 10 cm distance from the microphone according to their datasheet (see https://www.vybronics.com/coin‐vibration‐motors/lra/v‐g0832029d). The motors were housed in a PLA plastic plate (6 × 2.5 cm) attached to the skin with adhesive tape positioned immediately adjacent to the brushed area on the medial forearm. Similarly to the brushing duration, the vibration duration was also 4.4 s. The vibration plate was active exclusively during the “vibration” condition. In the “brushing” condition, the robotic arm made direct contact with the skin, applying the brush at CT‐optimal speeds. To ensure experimental consistency, the robotic arm executed identical bidirectional movements across all SEP conditions—first from proximal to distal, then from distal to proximal, in a continuous back‐and‐forth pattern. In the brushing conditions, the brush contacted the skin throughout both directional movements, during which seven electrical stimuli were delivered per movement phase. In contrast, in the “alone” and “vibration” SEP conditions, the robotic arm replicated the exact same bidirectional trajectories, but the brush was kept separate from the skin to avoid C‐tactile stimulation. This design aimed to maintain consistent auditory input and electrical noise generated by the activation of the robotic motors across all conditions.

Before the experiment, participants were instructed to adjust the vibratory stimulus's intensity to match the brushing's perceived intensity. They used a visual analog scale on a computer screen to increase or decrease the vibration intensity while the brushing speed and pressure remained constant. The final vibration intensity used during the experimental task was calculated as the average of three calibration trials.

Electrical Stimulation was administered using a Digitimer DS7A constant current stimulator (Digitimer) controlled by the Psychopy software (Peirce 2007). A surface stimulation electrode was placed on the left wrist crease (anode), with the cathode positioned 2 cm proximal to the wrist crease. Before the experiment, the intensity of the electrical stimulus was calibrated for each participant. The intensity was increased until the experimenter observed involuntary twitches of approximately 1 cm in fingers innervated by the median nerve (*M* = 14.1 mA; SD = 0.7 mA).

**FIGURE 1:**
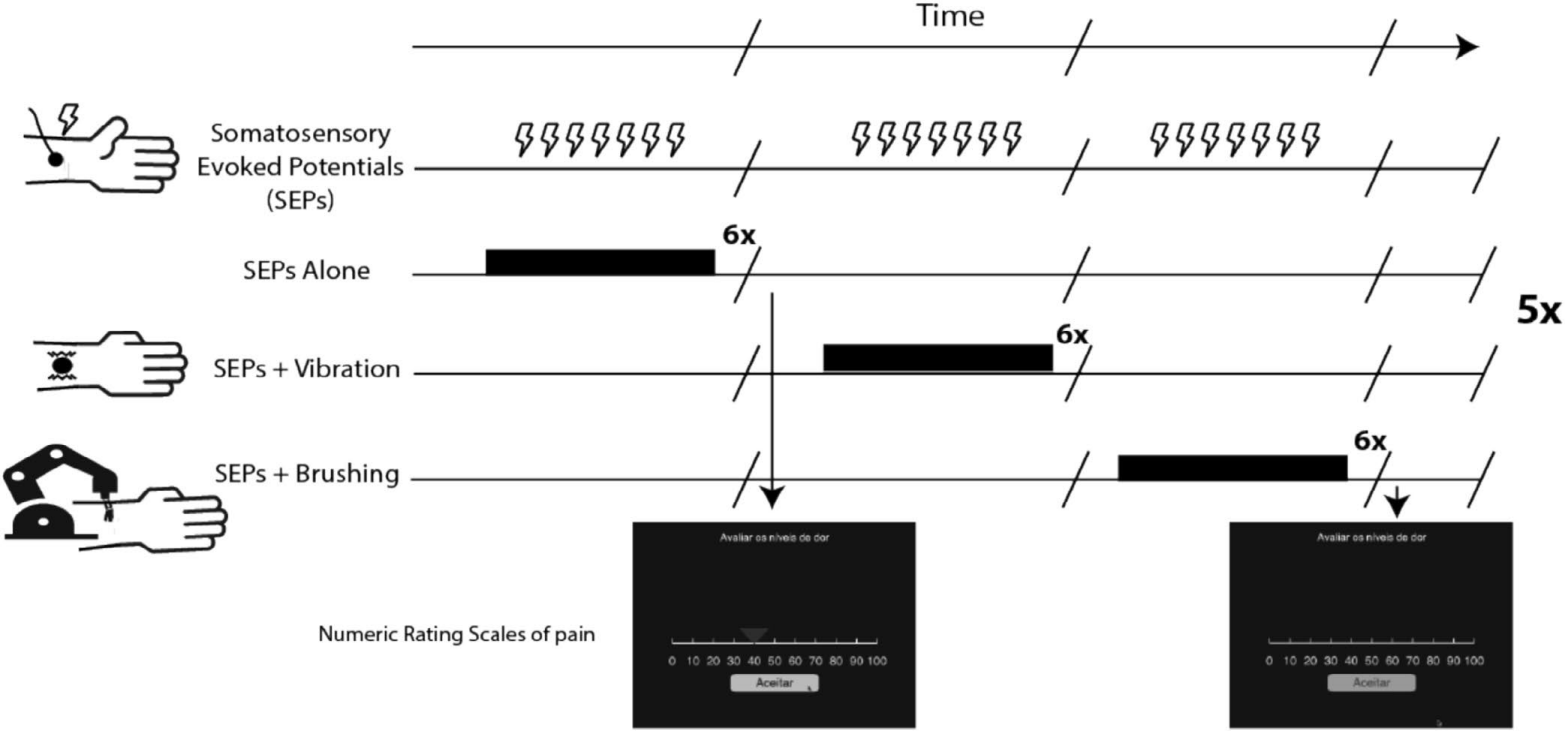
Visual description of the task. Electrical stimuli were applied to the median nerve to record Somatosensory Evoked Potentials (SEPs). These stimuli were presented during three experimental conditions: SEPs Alone, SEPs + Vibration (discriminative touch), and SEPs + Brushing (CT stimulation). For each condition block, six consecutive (6×) trains of seven electrical stimuli were presented, totaling 42 electrical stimuli per block. Each condition block was presented five times (5×) in a counterbalanced order across participants. Participants were asked to “Rate the pain level” (translation from: “Avaliar os níveis de dor”) produced by the electrical stimuli using a numeric rating scale at the end of some blocks.

Electrophysiological recording occurred in a soundproofed, electrically shielded room with controlled lighting. Participants wore headphones playing pink noise at 70 dB to ensure noise isolation. A total of 630 electrical pulses were delivered to the participants across five blocks per condition (making a total of 15 blocks). Each condition block comprised six trains of seven electrical stimuli. The seven‐stimulus‐long trains were presented during brushing (three trains during brushing in a proximal direction and the other three during brushing in a distal direction), during vibration, or without concomitant stimulation. The resulting electrical stimuli per condition were 210. The seven‐stimulus‐long trains were delivered at 2 Hz, with electrical pulses occurring at 0.7, 1.2, 1.7, 2.2, 2.7, 3.2, and 3.7 s following the onset of brushing or vibration when applicable. A brief pause (approximately 10 s) was included between each train to minimize fatigue and adaptation. The electrical stimulus intensity was defined individually during a calibration phase to achieve a consistent somatosensory experience across participants. The order of the blocks was randomized for each participant and fully counterbalanced across participants, with all six possible block orders repeated five times to form the 15 blocks. The six possible block orders were: [“Alone”, “Brushing”, “Vibration”]*5; [“Alone”, “Vibration”, “Brushing”]*5; [“Vibration”, “Brushing”, “Alone”]*5; [“Vibration”, “Alone”, “Brushing”]*5; [“Brushing”, “Alone”, “Vibration”]*5; [“Brushing”, “Vibration”, “Alone”]*5. After every two blocks, participants were instructed to rate the sensation produced by approximately the previous 10 electric stimuli on a numeric rating scale (NRS) with verbal anchors (0—“no sensation”; 10—“noticeable sensation”; 20—“mildly painful sensation” (i.e., pain threshold); 30—“very weak pain”; 40—“weak pain”; 50—“moderate pain”; 60—“slightly strong pain”; 70—“strong pain”; 80—“very strong pain”; 90—“nearly intolerable pain”; and 100—“unbearable pain”). To assist them with the pain assessment, they had a sheet of paper on top of the screen with “verbal anchors” for each value of the NRS. These ratings were collected twice per condition in the even‐numbered blocks leading up to block 12. For example, if the block order was: [“Alone”, “BRUSHING”, “Vibration”, “ALONE”, “Brushing”, “VIBRATION”, “Alone”, “BRUSHING”, “Vibration”, “ALONE”, “Brushing”, “VIBRATION”, “Alone”, “Brushing”, “Vibration”], participants had to rate the pain produced by electrical stimulation in the blocks highlighted in uppercase (Figure 1).

### 2.4 Electrophysiological Data

The electroencephalogram was recorded using a BioSemi ActiveTwo system (Biosemi, Amsterdam, the Netherlands). Participants wore a nylon cap fitted with 64 active Ag/AgCl scalp electrodes positioned according to the international 10–10 system. In addition to the scalp electrodes, four active electrodes were placed at the lateral canthi of both eyes (horizontal electrooculogram, HEOG), above and below the left eye (vertical electrooculogram, VEOG), and at the tip of the nose (used as a reference). The signals were referenced to the Common Mode Sense (CMS) and Driven Right Leg (DRL) electrodes from the BioSemi system.

Before recording, electrode offsets were confirmed to be below 30 mV. Electrophysiological signals were sampled at a rate of 512 Hz, with an online bandpass filter ranging from 0.01 to 100 Hz.

## 3 Data Analysis

### 3.1 EEG Processing

EEG data were analyzed using the EEGLAB v2023.1 toolbox for Matlab (R2022b). Segments containing major muscular or other artifacts were manually removed. To reduce artifacts caused by electrical stimulation, samples from −2 to 8 ms after stimulus onset were zeroed. Signals were filtered offline using a high‐pass FIR filter with a cut‐off frequency of 1 Hz, as implemented in EEGLAB's pop_eegfiltnew function. The filter was designed with an order of 1690, a transition band width of 1 Hz, a passband edge at 1 Hz, and a −6 dB cutoff at 0.5 Hz. Electrodes exhibiting poor recording quality were removed and reconstructed using spherical spline interpolation.

Epochs were extracted from continuous EEG data within a time window of −100 to 400 ms relative to the onset of electrical stimuli, with a baseline correction applied from −90 to −10 ms. Extended Infomax Independent Component Analysis (ICA) was used to correct ocular artifacts. Components associated with eye movements, muscle activity, and poor signal quality were rejected with the assistance of the EEGLab plug‐in “ICLabel” for component classification and removal. This process allows for the separation and elimination of eye‐related artifacts, improving the overall quality of the data.

The EEG data was re‐referenced to an average reference, and waveforms were computed by averaging all trials for each condition. Peak amplitudes for N20, P25, N30, and P45 were measured at electrode C4 (contralateral to the left hand). N20 and N30 were identified as the most negative (or less positive) peaks within the 15–20 and 30–40 ms windows, respectively, while P25 and P45 were the most positive (or less negative) peaks within the 20–30 and 35–50 ms windows following stimulus onset. N60 and P150 were measured at electrode FCz, with N60 identified as the most negative peak within the 40–100 ms window, and P150 as the most positive peak within the 100–200 ms window. As noted in previous studies, P150 reached its maximum amplitude at fronto‐central locations (Cruccu et al. 2008; Maudrich et al. 2022; Perri et al. 2019).

### 3.2 Statistical Analysis

Data analyses were conducted using JASP (version 0.18.3.0). One‐way repeated measures ANOVAs were performed to analyze Numeric Rating Scale (NRS) scores, which assessed perceived sensation and nociception, as well as electrophysiological data across the three experimental conditions: SEPs alone, SEPs with concomitant brushing, and SEPs with concomitant discriminative touch. Partial Eta Squared ($\eta_{p}^{2}$) was calculated to estimate effect sizes. Furthermore, Bayesian statistics were applied to enhance the reliability and interpretability of the results. Bayes factors (BF_10_) were computed to quantify evidence for both the alternative and null hypotheses. For the Bayesian repeated‐measures ANOVAs, we used Cauchy priors on effect sizes with a scale parameter of 0.5 for fixed effects and 1 for random effects, following the recommendations of Rouder et al. (2009) and Morey and Rouder (2011). Model priors were set to uniform, indicating no prior preference for any specific model. For the Bayesian correlation analyses, a stretched beta prior on the correlation coefficient was applied with a prior width of 1.0, as proposed by Ly et al. (2016).

Post hoc paired comparisons were adjusted for multiple comparisons using Holm correction, with effect sizes reported as Cohen's *d*. Pearson correlations were used for normally distributed data, while Spearman correlations were employed for non‐normally distributed data to assess associations between psychological measures, pain ratings, and SEPs. The correlation analysis included 26 participants due to missing data on the questionnaires because of technical errors. As no specific hypotheses regarding the direction of correlations were formulated, False Discovery Rate (FDR) correction for multiple comparisons was applied (Benjamini and Yekutieli 2001).

## 4 Results

### 4.1 Somatosensory Evoked Potentials (SEPs)

No condition effect was observed for any of the SEPs components measured over electrode C4, including N20 (*F*_(2,58)_ = 0.32; *p* = 0.73; $\eta_{p}^{2}$ = 0.01; BF_10_ = 0.13), P25 (*F*_(2,58)_ = 1.06; *p* = 0.35; $\eta_{p}^{2}$ = 0.04; BF_10_ = 0.23), N30 (*F*_(2,58)_ = 1.75; *p* = 0.18; $\eta_{p}^{2}$ = 0.06; BF_10_ = 0.38), and P45 (*F*_(2,58)_ = 1.39; *p* = 0.26; $\eta_{p}^{2}$ = 0.05; BF_10_ = 0.29), indicating substantial evidence against the presence of any difference between conditions (see data in Table 1). Given that these early components exhibit centroparietal topographies, we also analyzed them at electrode CP4 and found no condition effect for any measured components.

**TABLE 1:** Descriptive statistics.

Measure	SEPs + Brushing	SEPs + Vibration	SEPs Alone
NRS	33.383 (22.408)	33.55 (21.211)	33.067 (22.965)
N20 (C4)	−0.249 (0.581)	−0.212 (0.546)	−0.178 (0.60)
P25 (C4)	1.535 (1.382)	1.682 (1.437)	1.631 (1.411)
N30 (C4)	0.28 (1.212)	0.445 (1.180)	0.456 (1.348)
P45 (C4)	2.049 (1.420)	2.215 (1.568)	2.166 (1.532)
N60 (FCz)	−2.625 (1.298)	−2.563 (1.392)	−2.474 (1.332)
P150 (FCz)	2.021 (1.261)	2.472 (1.571)	2.616 (1.679)

*Note:* Mean (standard deviation in parentheses) of reported pain in the Numeric Rating Scale (NRS) and amplitudes (in μV) of the peaks of the Somatosensory Evoked Potentials (SEPs) at the analyzed electrodes (C4 and FCz) in each of the three experimental conditions.

**FIGURE 2:**
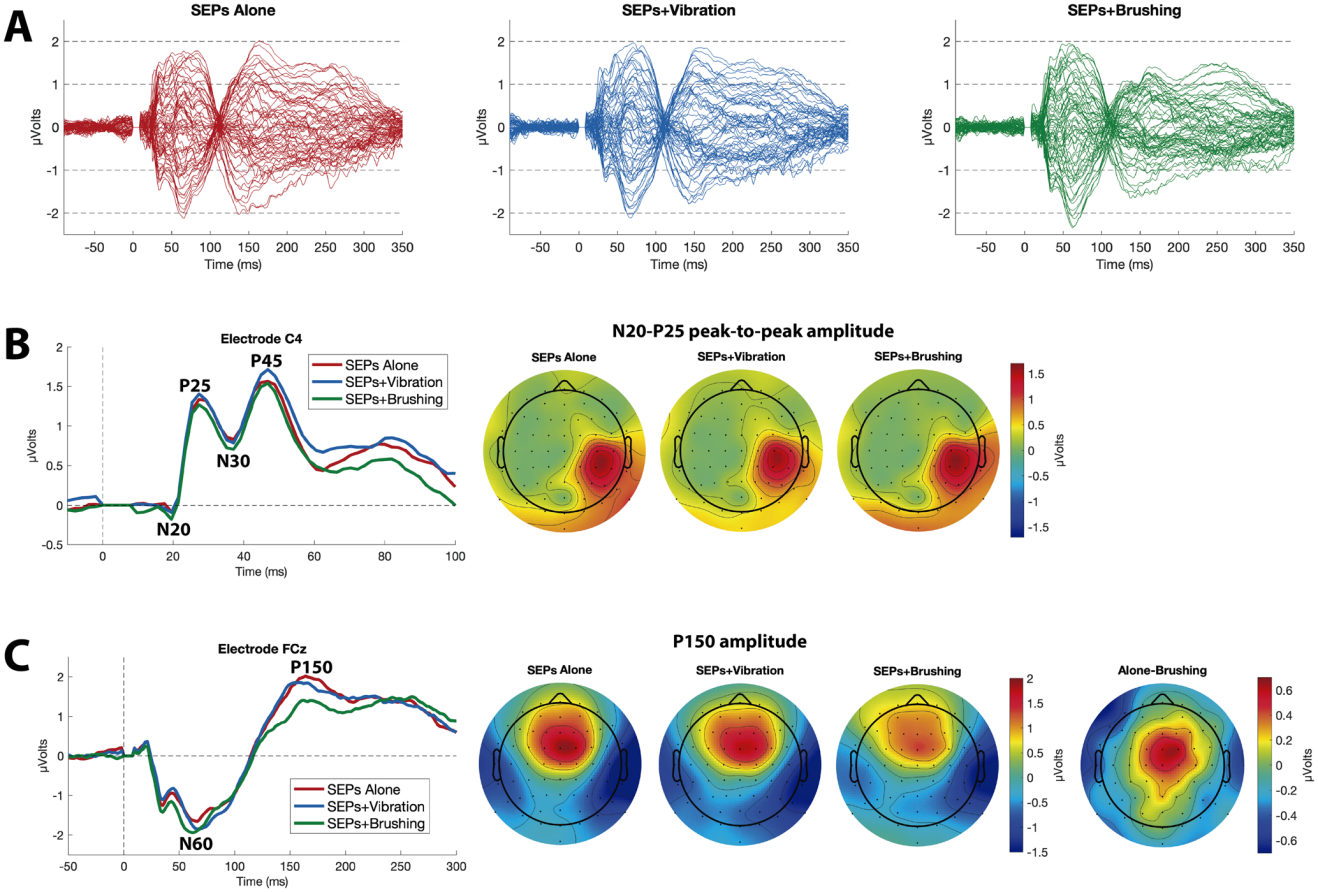
(A) Butterfly plot of the event‐related activity time‐locked to the presentation of the electrical stimuli, recorded in all the scalp electrodes and for each condition. (B) Somatosensory evoked potentials (SEPs) recorded over the C4 electrode (N20, P25, N30, and P45) and topographies of the N20–P25 peak‐to‐peak amplitudes under the different experimental conditions; (C) Event‐related activity time‐locked to the presentation of the electrical stimuli recorded over the FCz electrode (N60 and P150) and topographies of the P150 component under different experimental conditions: SEPs alone, SEPs with concomitant discriminative touch, and SEPs concomitant with brushing. The final topography (at the bottom right) shows the difference between SEPs alone and SEPs with brushing.

In contrast, for the components measured over electrode FCz, a significant effect of Condition was found for the P150 amplitude (*F*_(2,58)_ = 6.39; $\eta_{p}^{2}$ = 0.18; *p* = 0.003; BF_10_ = 11.33), indicating a moderate effect size and strong evidence supporting the presence of differences between conditions. Post hoc comparisons revealed differences between SEPs+Brushing versus SEPs Alone (*t*_(29)_ = −3.43; *p* = 0.003; *d =* −0.393; BF_10_ = 6.49) and SEPs+Brushing versus SEPs+Vibration (*t*_(29)_ = −2.60; *p* = 0.02; *d =* −0.298; BF_10_ = 5.51), indicating moderate effect sizes. No difference was observed between SEPs+Vibration versus SEPs Alone (*t*_(29)_ = −0.83; *p* = 0.41; *d = −*0.095; BF_10_ = 0.29) (Figure 2). While a high‐pass cutoff of approximately 1 Hz (as the one used here) is recommended and commonly used in SEP studies (e.g., Colon and Comi 1990, 295; MacDonald et al. 2019), these filters can cause distortions in evoked activity, and lower high‐pass cutoff filters are recommended for general event‐related potentials studies (Luck 2014). To address potential concerns regarding signal distortion from the 1 Hz high‐pass filter, we reanalyzed the data using a less restrictive 0.25 Hz filter. The same pattern of significant effects was observed, confirming the P150 findings (see Figure S1 for the lower high‐pass filtered data).

No condition effect was found for the N60 component (*F*_(2,58)_ = 0.55; *p* = 0.58; $\eta_{p}^{2}$ = 0.02; BF_10_ = 0.15), providing strong evidence against the existence of differences between conditions. We also analyzed N60 and P150 in the FC2 electrode, which exhibited the next highest amplitude after FCz. The findings were replicated in this electrode, and the same significant comparisons were obtained for FCz.

### 4.2 Correlation Between SEPs and Psychological Questionnaires

**FIGURE 3:**
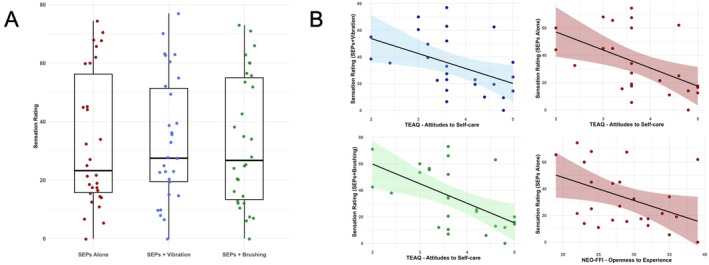
(A) Distribution of sensation ratings across experimental conditions: SEPs Alone, SEPs with Vibration, and SEPs with Brushing (each dot represents the mean value for each participant). (B) Correlations between subjective sensation ratings and self‐report measures (with the shaded area representing the 95% confidence interval): the sensation rating for the SEPs with Vibration condition and the Attitudes to Self‐Care subscale of the TEAQ (upper left); the sensation rating for the SEPs Alone condition and the Attitudes to Self‐Care subscale of the TEAQ (upper right); the sensation rating for the SEPs with Brushing condition and the Attitudes to Self‐Care subscale of the TEAQ (lower left); the sensation rating for the SEPs Alone condition and Openness to Experience from the NEO‐FFI (lower right).

Exploratory correlations between the P150 SEP component and self‐reported psychological measures or the NRS ratings were not significant (see distribution in Figure 3). Initially, a negative correlation was observed between Attitude to Self‐Care (from the TEAQ) and subjective sensation ratings across the three conditions: SEPs+Brushing (*r* = −0.56, *p* = 0.003; BF_10_ = 17.47); SEPs+Vibration (*r* = −0.46, *p* = 0.018; BF_10_ = 3.49); and SEPs Alone (*r* = −0.51, *p* = 0.007; BF_10_ = 7.46). Additionally, Openness to Experience (from the NEO‐FFI) initially correlated with subjective sensation ratings in the SEPs Alone condition (*r* = −0.48; *p* = 0.03; BF_10_ = 2.29) (see Figure 3B). Although there was moderate to strong evidence of the correlation between the sensation ratings and Attitude to Self‐Care (TEAQ), none of the correlations survived the False Discovery Rate (FDR) correction, suggesting the initial findings may have been due to chance after correcting for multiple comparisons.

## 5 Discussion

C‐tactile (CT) afferents have been shown to modulate nociceptive stimuli, but their role in modulating other somatosensory inputs remains unclear. The primary objective of this study was to determine whether CT stimulation can modulate somatosensory afferent activity in the central nervous system. To explore this, we compared somatosensory evoked potentials (SEPs) during three conditions: SEPs with concomitant CT‐targeted stimulation (brushing), SEPs with discriminative touch (vibration), and SEPs alone (no touch). While no significant differences were found between conditions in early‐latency SEPs (N20, P25, N30, and P45), we observed a lower amplitude of the P150 component during CT‐targeted stimulation (brushing) compared to the other conditions. No difference was found between SEPs alone and SEPs with vibration.

These findings suggest that CT afferents, which are activated by brushing, modulate somatosensory processing in ways that differ from A‐beta fiber activation, as the vibration condition only stimulated A‐beta fibers without CT involvement.

Earlier components reflect distinct stages of somatosensory processing and are often reported in studies utilizing median nerve stimulation, differing from posterior components (Maudrich et al. 2022). The N20 and P25 components are presumably generated by neurons in the anterior wall of the postcentral gyrus (S1) (Peterson et al. 1995). The N30 component has been attributed to different neural sources, with ongoing debate regarding its origin. Some studies suggest that it reflects somatosensory activity, either as the negative counterpart of the parietal P30 generated in area 3b (Allison et al. 1991; Bötzel et al. 1995) or as a summation of responses within the somatosensory areas (Valeriani et al. 2000). Others propose that it arises from motor‐related regions, such as the supplementary motor area (SMA) and dorsal premotor cortex (Valeriani et al. 2000). Similarly, the P45 component has been recorded in the precentral gyrus (Allison et al. 1989) and N60 in SMA and S1 (Barba et al. 2005). Our study's absence of differences in these early SEP components across conditions suggests that early somatosensory cortical processing may not be modulated by CT stimulation. Previous studies have shown that vibratory stimulation of the hands and fingers interferes with early SEP components (Jones 1981; Jones and Power 1984); these discrepancies with our results can be explained by a lower density of discriminative touch receptors in the forearm (Vallbo et al. 1999; Corniani and Saal 2020). Similarly, light tactile stimulation has been reported to influence early neural responses in frontal and central‐parietal components during contralateral forearm stimulation (Kakigi and Jones 1985). Inconsistencies with our results may be due to methodological differences. In the Kakigi and Jones (1985) study, the design of the light tactile stimulation did not specifically target CT fibers. Additionally, the researchers employed a group average difference waveform analysis—subtracting the interfering tactile stimuli condition from the control (SEPs‐only) condition—and did not report statistical results.

A methodological consideration of our study is the use of a seven‐stimulus train‐based stimulation arrangement. While designed for our experimental protocol, this approach may introduce slow waveform shifts related to fluctuating attention or expectation across the trains. To clarify this potential issue, in Figure S1, we plotted the neural activity evoked by the brushing or vibration onset. Visual inspection suggests that it largely returned to baseline before the first electrical stimulus; nevertheless, the limited trial count and inherent signal‐to‐noise ratio for these specific time‐locked responses do not allow for clear conclusions about the stability of the pre‐stimulus baseline. Therefore, the potential influence of such slow drifts should be considered when interpreting the P150 results.

In sum, at the psychophysiological level, we observed no differences between the different stimulation conditions in these SEPs, which could suggest that early cortical processing of somatosensory information is not affected by CT stimulation.

In contrast, the reduced amplitude of the P150 component in the brushing condition points to the potential influence of attentional and cognitive factors on later stages of somatosensory processing (Cruccu et al. 2008; Niso et al. 2021). The P150 component, often linked to conscious perception of somatosensory stimuli, has been localized in the anterior cingulate cortex (ACC) and the right anterior insula (Gordon et al. 2013; Perri et al. 2019; Shackman et al. 2011), regions associated with both pain and affective touch processing. The processing of these two modalities follows similar patterns with juxtaposed roles in motivational functions and opposite affective valences (negative and positive) (Holmes 2023). These findings suggest that CT stimulation may influence sensory processing by engaging brain regions responsible for motivational and affective functions, potentially modulating attention or socio‐cognitive aspects of touch.

The observed reduction in P150 amplitude during CT‐targeted stimulation might also be attributed to a decrease in arousal, reflecting the soothing, stress‐reducing effects of CT‐optimal touch (Harlow 1959; Sharp et al. 2012). Another possibility is that CT‐targeted stimulation diverted attention away from the electrical stimuli, leading to a diminished P150 response. In this sense, other later components, such as N140, were reported as sensitive to focused attention, exhibiting increased amplitude for attended stimuli (Kida et al. 2023; García‐Larrea et al. 1995). Other studies have shown that the N140 SEP can also be modulated independently of attentional allocation in specific contexts, such as during tasks involving hand movement, suggesting that it may also reflect motor preparation and somatosensory filtering mechanisms associated with movement execution (Kida et al. 2004, 2006; Nakata et al. 2003). The fact that the vibration condition, which also engaged somatosensory pathways but without CT‐targeted stimulation, did not produce the same effect suggests that CT stimulation's salience may arise from its social and affective nature. This supports the notion that CT stimulation engages neural circuits related to social cognition and emotional regulation, reinforcing the importance of touch in human bonding and communication (McGlone et al. 2014; Morrison 2016b). Finally, it is also possible that the observed reduction in P150 was not only due to CT stimulation since brushing can partially activate the A‐beta receptors, and these afferents might interact at the dorsal horn level and modulate signaling (Schirmer et al. 2022).

Previous research has demonstrated that interpersonal touch can reduce perceived pain levels (Goldstein et al. 2016; Saarinen et al. 2021; Savallampi et al. 2023) and cortical responses evoked by phasic nociceptive stimuli (Von Mohr, Krahé, et al. 2018). For instance, Fidanza et al. 2021, found that gentle touch reduced temporal summation of second pain (TSSP), a model of central pain sensitization. Clinical studies have also shown that affective touch can alleviate chronic pain (Meijer et al. 2023). However, in our study, subjective pain ratings were not significantly affected by the experimental conditions, and no clear relationship between pain ratings and brain activity indices or self‐report measures emerged. This suggests that basic pain indices may not fully capture the complex interplay of experiences and attitudes toward touch, as measured by the TEAQ.

A critical aspect of our study was that CT‐targeted stimulation was delivered using a robotic brush, eliminating the socio‐emotional components inherent in natural interpersonal touch. Whether the results would have differed with human stroking remains unclear, though previous studies have linked slow brushing to pain modulation in other contexts (Habig et al. 2017; Liljencrantz et al. 2017). Another potential reason for the lack of significant differences in subjective ratings could be that all participants did not perceive the electrical stimuli as painful despite being somewhat threatening or arousing (Perri et al. 2019). The electrical stimulation used for obtaining somatosensory evoked potentials mainly activates axons that convey mechanoreceptive and proprioceptive information via the dorsal column–medial lemniscus pathway. Thus, our findings suggest that CT stimulation not only soothes the nociceptive volley of the spinothalamic tract (as reported in previous research) but also affects mechanoreception conveyed by large‐diameter nerve fibers of the lemniscal system (Cruccu et al. 2008; MacDonald et al. 2019).

A limitation of this study is that the pleasantness of the brushing was not controlled across participants. CT fiber activity correlates with perceived pleasantness, and variations in pleasantness could influence how CT stimulation modulates somatosensory input (Ackerley et al. 2014; Liljencrantz et al. 2017).

Future research could benefit from examining earlier SEP components, such as N13, to further understand how CT stimulation affects spinal‐level mechanisms involved in pain modulation. Additionally, using human touch instead of robotic stimulation could provide insights into whether naturalistic affective touch has a stronger impact on somatosensory processing, particularly in top‐down modulation. Including a separate condition without electrical stimulation while maintaining identical brushing or vibration parameters could also establish a more robust baseline for comparison and enhance the interpretability of the findings.

## 6 Conclusion

This study provides evidence that slow brushing, which activates CT‐optimal fibers, modulates somatosensory input, as reflected by the reduced amplitude of the P150 component in fronto‐central regions. This suggests that CT‐optimal touch influences not only nociceptive processing but also mechanoreception via the lemniscal system. The attenuation of the P150 component may indicate engagement of brain regions involved in attentional, cognitive, and socio‐emotional processing. These findings highlight the complex role of affective touch in sensory modulation. Further research should investigate the effects of naturalistic human touch and the potential involvement of earlier somatosensory processing stages in CT stimulation.

## Author Contributions

**A. Ribeiro‐Carreira:** conceptualization, data curation, formal analysis, investigation, methodology, validation, visualization, writing – original draft. **Márcia da‐Silva:** investigation, writing – review and editing. **Ana Rita Pereira:** investigation, writing – review and editing. **Maria Teresa Carrillo‐de‐la‐Peña:** writing – review and editing. **Joana Coutinho:** writing – review and editing. **Adriana Sampaio:** resources, supervision, writing – review and editing. **Alberto J. González‐Villar:** conceptualization, data curation, formal analysis, funding acquisition, methodology, project administration, resources, software, supervision, validation, writing – review and editing.

## Conflicts of Interest

The authors declare no conflicts of interest.

## Supporting information


**FIGURE S1:** Photograph of the experimental setup. The black arrows represent the direction of the brush's movement, while the red flashes represent the moments of electrical stimulation during the movement of the brush (note that the electrical stimulation electrode has not been placed for this picture). The black plate taped to the arm houses the vibrating motors.
**FIGURE S2:** ERPs at the FCz electrode using a 0.25 Hz high‐pass filter (cutoff frequency: 0.125 Hz). Analysis of the P150 component replicated the main findings: Main effect of condition: *F*(2, 58) = 8.61, $\eta_{p}^{2}$ = 0.23, *p* < 0.001; Post hoc comparisons: SEPs+Brushing versus SEPs Alone: *t*(29) = −4.12, *p* < 0.001; SEPs+Brushing versus SEPs+Vibration: *t*(29) = −2.45, *p* = 0.031. These results suggest that the observed effects are not attributable to filtering artifacts.
**FIGURE S3:** Train‐level ERPs time‐locked to the onset of the electrical stimulation trains. Time = 0 corresponds to the presentation of the first electrical stimuli of the block, therefore the vibration or the brushing starts from around −0.7 s. Vertical lines every 0.5 s are the artifacts produced by the electrical stimulation.

## Data Availability

The EEG, behavioral data, and code used are available upon request to the corresponding author, with the condition that compliance with ethical procedures governing the reuse of the data must be maintained.
