# Supplementary material for: CT‐Optimal Stimulation Modulates Somatosensory Processing
Source: Psychophysiology. 2025 Sep 8;62(9):e70146. doi: 10.1111/psyp.70146 (PMC12415938; doi:10.1111/psyp.70146)

Supplementary Material

Supplementary Figure 1. Photograph of the experimental setup. The black arrows represent the direction of the brush's movement, while the red flashes represent the moments of electrical stimulation during the movement of the brush (note that the electrical stimulation electrode has not been placed for this picture). The black plate taped to the arm houses the vibrating motors.

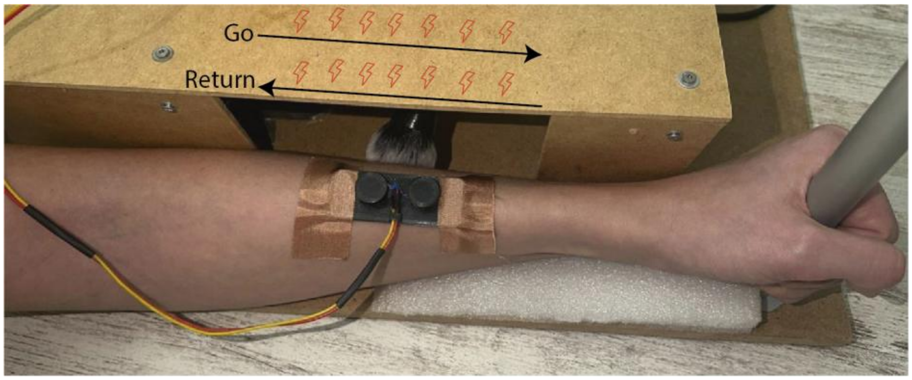

Supplementary Figure 2. ERPs at the FCz electrode using a 0.25 Hz high-pass filter (cutoff frequency: 0.125 Hz). Analysis of the P150 component replicated the main findings: Main effect of condition: $F(2,58) = 8.61$, $\eta^2_p = 0.23$, $p < .001$; Post-hoc comparisons: SEPs+Brushing vs. SEPs Alone: $t(29) = –4.12$, $p < .001$; SEPs+Brushing vs. SEPs+Vibration: $t(29) = –2.45$, $p = .031$. These results suggest that the observed effects are not attributable to filtering artifacts.

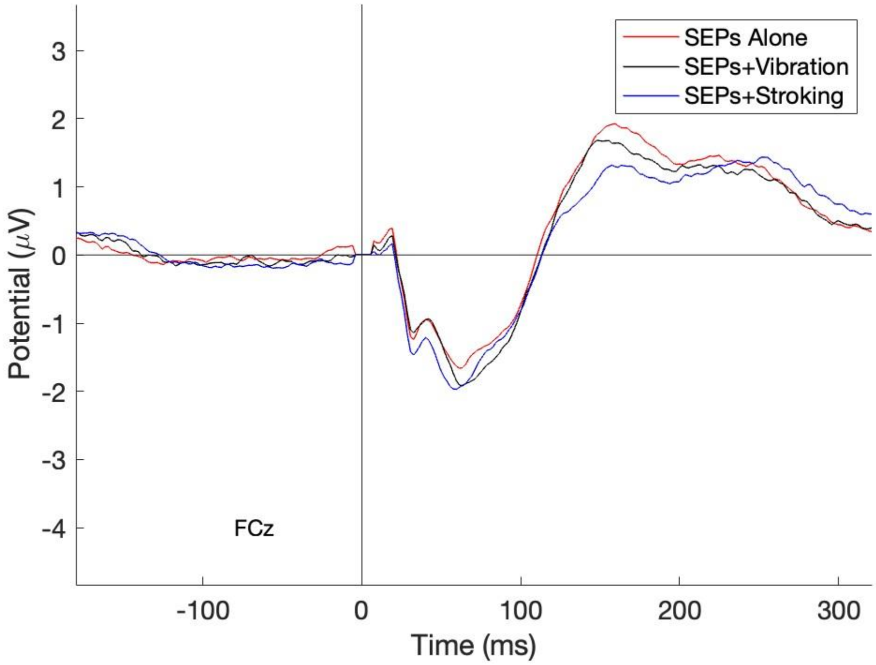

Supplementary Figure 3. Train-level ERPs time-locked to the onset of the electrical stimulation trains. Time = 0 corresponds to the presentation of the first electrical stimuli of the block, therefore the vibration or the brushing starts from around -.7 seconds. Vertical lines every 0.5 s are the artifacts produced by the electrical stimulation.

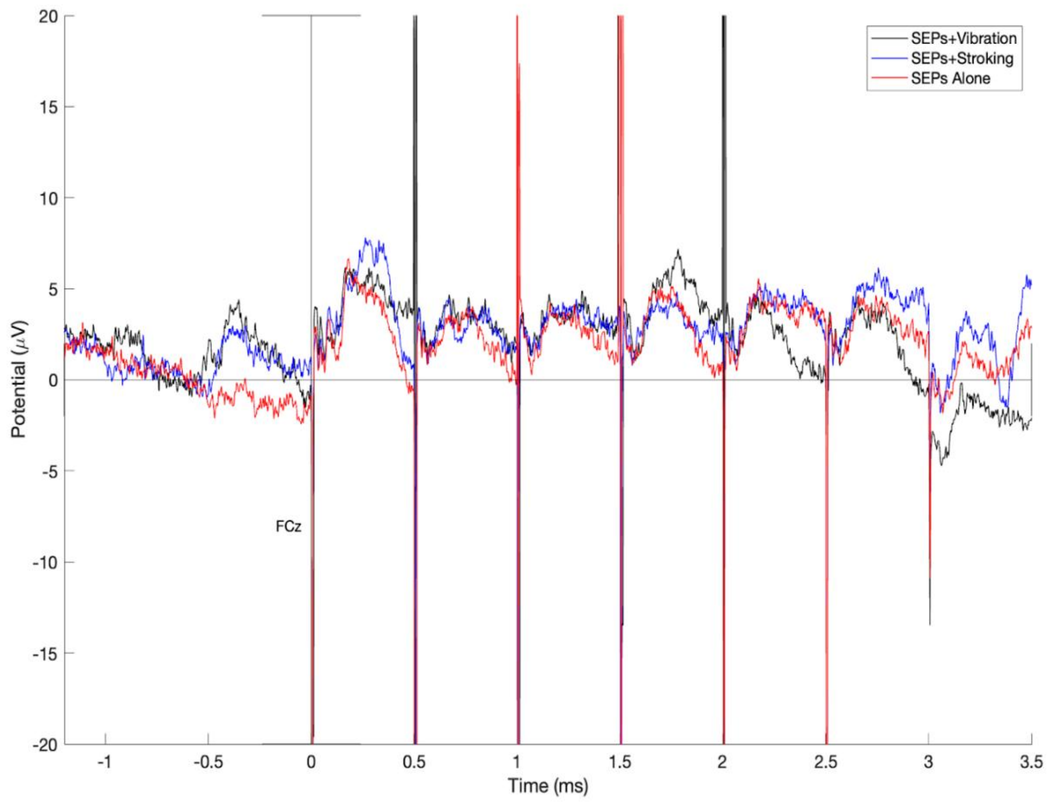

Supplement: Supplementary file 1 — FIGURE S1: Photograph of the experimental setup. The black arrows represent the direction of the brush's movement, while the red flashes represent the moments of electrical stimulation during the movement of the brush (note that the electrical stimulation electrode has not been placed for this picture). The black plate taped to the arm houses the vibrating motors. FIGURE S2: ERPs at the FCz electrode using a 0.25 Hz high‐pass filter (cutoff frequency: 0.125 Hz). Analysis of the P150 component replicated the main findings: Main effect of condition: F(2, 58) = 8.61, ηp2 = 0.23, p < 0.001; Post hoc comparisons: SEPs+Brushing versus SEPs Alone: t(29) = −4.12, p < 0.001; SEPs+Brushing versus SEPs+Vibration: t(29) = −2.45, p = 0.031. These results suggest that the observed effects are not attributable to filtering artifacts. FIGURE S3: Train‐level ERPs time‐locked to the onset of the electrical stimulation trains. Time = 0 corresponds to the presentation of the first electrical stimuli of the block, therefore the vibration or the brushing starts from around −0.7 s. Vertical lines every 0.5 s are the artifacts produced by the electrical stimulation. [file PSYP-62-e70146-s001.pdf]
